# Novel Combination Therapy With Lenalidomide and Trametinib for Treatment-Resistant Xanthoma Disseminatum With Disabling Cutaneous and Synovial Involvement: A Case Report

**DOI:** 10.7759/cureus.84583

**Published:** 2025-05-21

**Authors:** Sundeep Bekal, Haifaa Abdulhaq

**Affiliations:** 1 Internal Medicine, Community Hospitalist Physician Group, Fresno, USA; 2 Hematology and Oncology, University of California San Francisco, Fresno Medical Education Center, Fresno, USA

**Keywords:** lenalidomide, mek inhibitor, non-langerhans cell histiocytosis, trametinib, xanthoma

## Abstract

Xanthoma Disseminatum (XD) is a rare histiocytic myeloid neoplasm involving the skin, mucosa, and sometimes the pituitary gland, and shares histologic features with juvenile xanthogranuloma (JXG). Given its rarity, there are no standardized treatment guidelines for XD, and management decisions are largely based on case reports and clinical judgment.

We present the case of a patient with XD who had extensive, disfiguring cutaneous lesions and disabling synovial involvement of the hips and knees with secondary bone erosions. The patient was initially treated with three separate lines of therapy, cladribine, cobimetinib, and clofarabine, all of which were discontinued due to either a lack of rapid clinical response or significant toxicity.

Due to both a lack of rapid response and toxicities, his therapeutic regimen was changed to reduced-dose trametinib along with lenalidomide. This combination led to a marked clinical and radiographic improvement across all affected sites. To our knowledge, this represents the first reported case of successful treatment of XD using a combination of a MEK inhibitor and lenalidomide.

## Introduction

Xanthoma disseminatum (XD) is a rare cutaneous non-Langerhans cell histiocytosis (n-LCH) with fewer than 200 cases reported in the literature. Histiocytoses are rare disorders characterized by the abnormal accumulation of histiocytes, a type of immune cell, in various tissues. XD is particularly challenging due to its unpredictable clinical course, potential for disfiguring and disabling involvement, and the absence of standardized treatment guidelines, given its rarity [1, 2]. Predominantly affecting young adult males under the age of 25, XD is characterized by the onset of small, yellow-red papules distributed on the face, trunk, and limbs, with significant intertriginous region involvement being common [3]. Key histologic features include S100-histiocytic proliferation, foam cells, and large multinucleated cells called Touton giant cells. Mutations in genes in the RAS-RAF pathway and anaplastic lymphoma kinase (ALK) pathway are often found, but are not required for diagnosis. Extracutaneous manifestations of XD involving the lung and central nervous system (particularly the pituitary gland) occur less frequently. While nonmalignant xanthogranulomatous changes of synovium, particularly tenosynovial giant cell tumors (also known as pigmented villonodular synovitis), have been well characterized, synovial XD has been reported only five times before [4-8].

Due to its rarity, there are no established management guidelines, and treatment is typically based on anecdotal evidence and individual case reports. Treatment choice and sequence are challenging. Time to response and depth of response are variable [9]. 

Recent studies have shown that somatic mutations that activate the mitogen mitogen-activated protein kinase (MAPK/ERK) pathway are detectable in most patients with Langerhans cell histiocytosis (LCH) and n-LCH, with BRAF being the most commonly mutated [10-12].

MEK inhibitors are a class of medications that block the mitogen-activated protein kinase kinase enzymes MEK1 and/or MEK2, which help control cell growth and survival. In fact, MEK inhibitors have been utilized in the treatment of malignant melanomas. Importantly, one MEK inhibitor, cobimetinib, was shown to be efficacious regardless of the specific MAPK/ERK mutations the patients possessed [13].

Trametinib is another MEK inhibitor approved for BRAF V600-mutant melanoma, non-small cell lung cancer, and anaplastic thyroid cancer, and has shown activity in MAPK-driven histiocytic neoplasms, including hairy cell leukemia. As opposed to cobimetinib’s intermittent dosing schedule, trametinib is taken as a daily dose by mouth for cancer therapy. In a study by Aaroe et al., 26 patients with non-Langerhans histiocytosis received trametinib. The response rate in the 17 evaluable patients was 71% (with 73% (8/11) without a detectable BRAF V600E achieving response) [14]. 

In addition to MEK inhibitors, immunomodulatory agents have activity against histiocyte disorders. One medication, thalidomide, has been approved to treat a number of cancers and skin disorders. While it has proved effective in low-risk, mucocutaneous, and/or bone forms of Langerhans cell histiocytosis, it is known to cause neurological adverse reactions of sedation and neuropathy [15,16]. 

Possessing improved potency and reduced side effects, lenalidomide is a functional and structural analog of thalidomide. Lenalidomide is well known to stop tumor proliferation through the induction of apoptosis [17]. Recent case reports have shown its efficacy in the treatment of histiocytic disorders, including Rosai-Dorfman disease [18]. In addition, lenalidomide’s use as a treatment for Langerhans cell histiocytosis with vulvar involvement has also been described in the literature [19]. 

## Case presentation

A 24-year-old male first noted “bumps” resembling warts on his axilla bilaterally three years prior to presentation. The patient reported that the lesions progressively spread over the following years, affecting his right inner thigh, perineum, and around his mouth. The patient’s initial skin lesions were believed to be acne vulgaris, and he was prescribed isotretinoin without improvement. He was previously healthy and did not have a contributory family history. 

**Figure 1:**
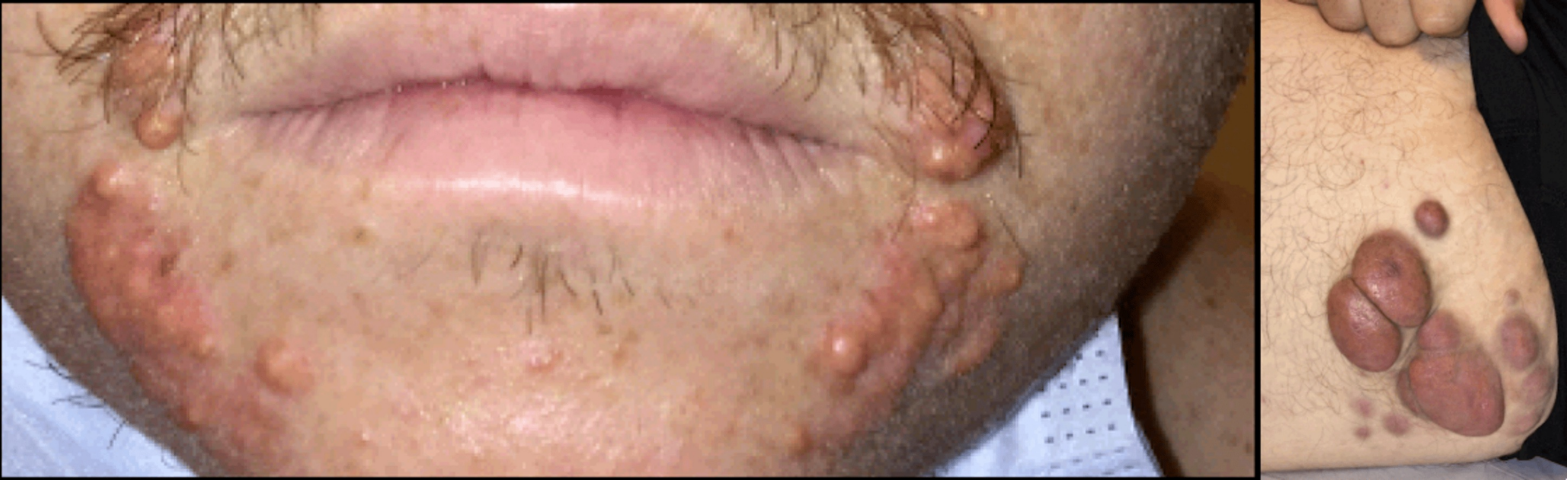
Initial lesions on face and right inner thigh

**Figure 2:**
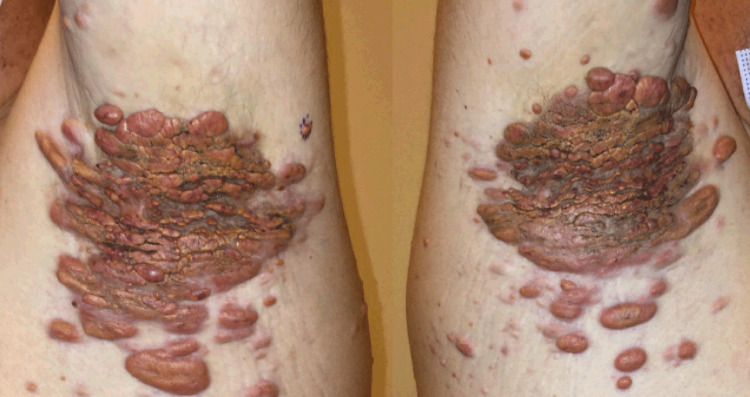
Initial lesions on bilateral axilla

Approximately six months later, he presented again with scattered diffuse erythematous orange-yellow papules, nodules, and plaques on the eyelids, perioral skin, bilateral axillae, trunk, perineum, and extremities (Figures 1, 2).

He also endorsed bilateral hip and knee pain, which was not resolved with over-the-counter medications. He had no symptoms of pituitary disease, and a brain MRI was normal. CBC with differential was normal. 

**Figure 3:**
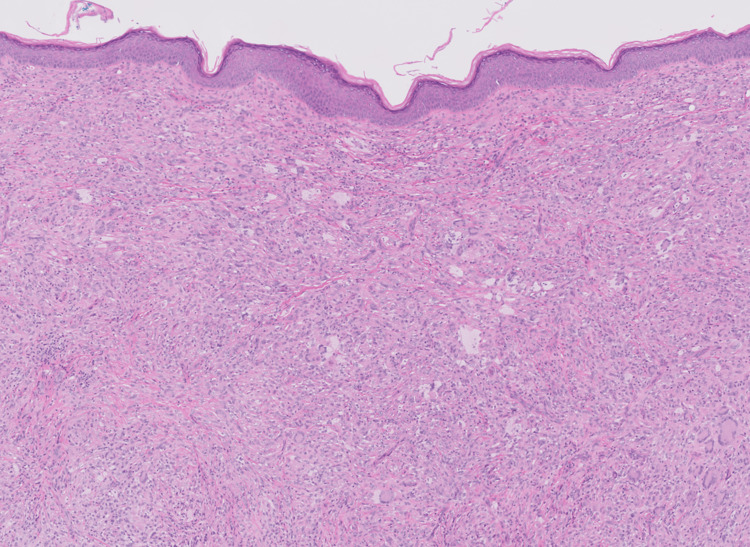
H&E (hematoxylin and eosin) stain showing dermal proliferation of histiocytes (with scattered Touton giant cells compatible with xanthogranuloma)

**Figure 4:**
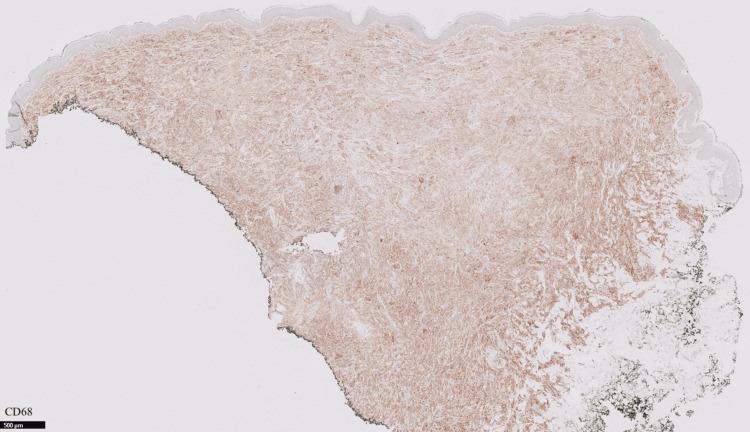
Positive CD68 stain

**Figure 5:**
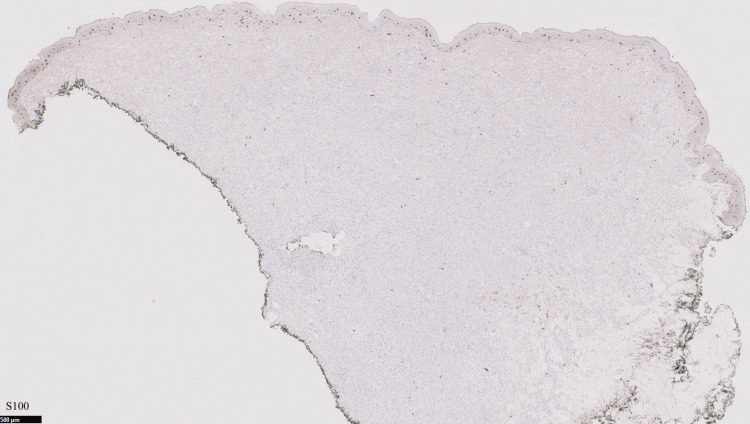
Negative S100 stain

**Figure 6:**
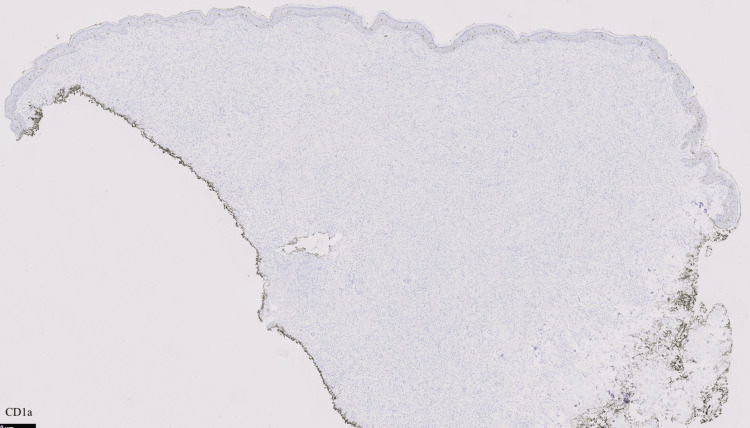
Negative CD1a stain

The patient had a punch biopsy obtained from his left axillary lesion, which revealed findings compatible with a xanthogranulomatous disease “CD68+, S100-, CD1a- histiocytic proliferation in the dermis with scattered Touton giant cells” (Figures 3-6).

**Figure 7:**
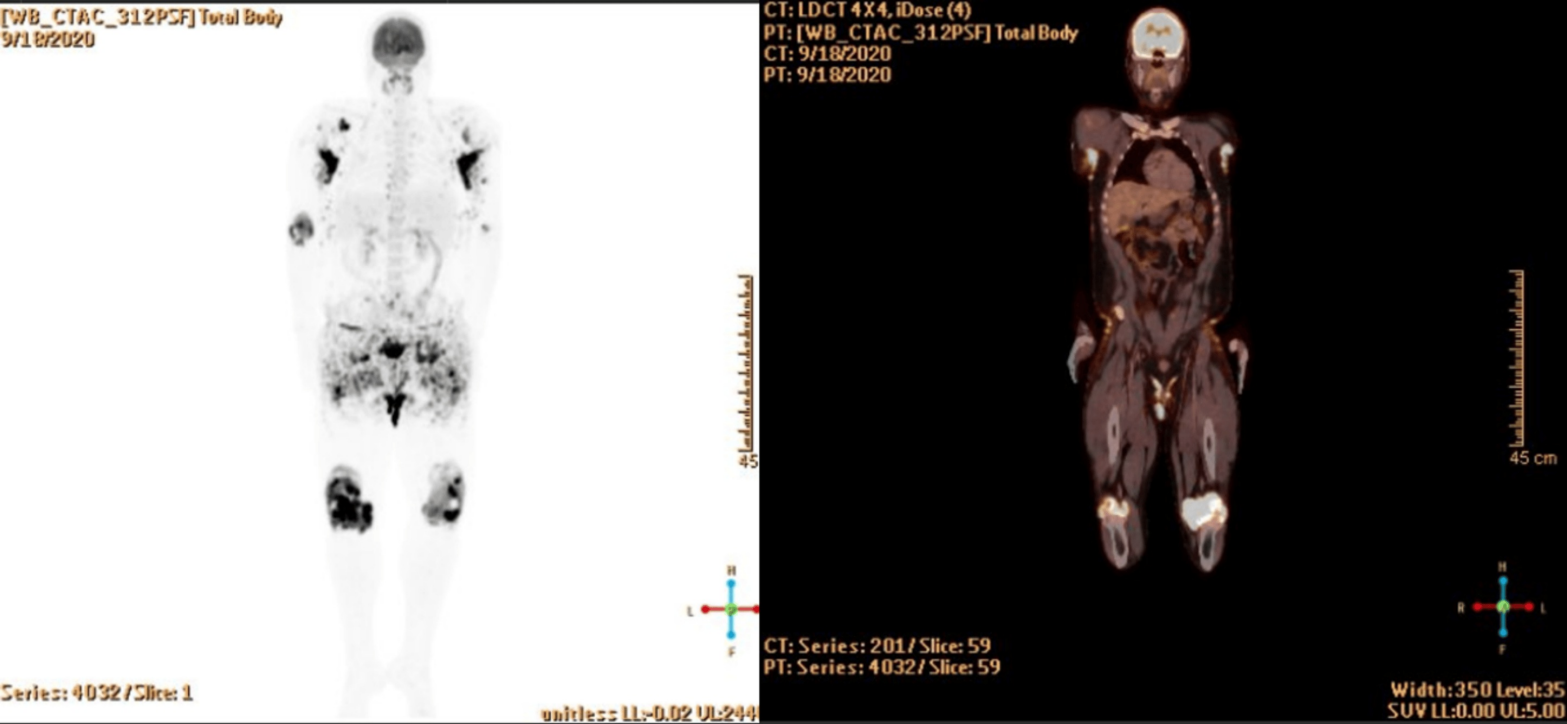
Hypermetabolic areas involving multiple joints, particularly in the knees

Due to the persistent symptoms and concern for joint involvement of his systemic histiocytosis, an FDG positron emission tomography-computed tomography (PET-CT) scan was ordered, which showed hypermetabolism involving multiple joints, particularly in the bilateral elbow/shoulders (SUVmax 27.5), bilateral hip joints (SUVmax 20.3). PET findings also commented on associated erosive changes, synovial thickening, and effusions in the knees bilaterally, with an SUVmax 34.8. There were also hypermetabolic cutaneous lesions throughout the body, but no metabolic evidence of central nervous system, pituitary, or any other organ involvement (Figure 7).

After a trial of corticosteroids did not improve joint pain, the patient was started on cladribine at 0.09 mg/kg/day IV for five days every three weeks. Despite maximal antiemetic regimens, he experienced grade 3 nausea and vomiting as defined by the Common Terminology Criteria for Adverse Events (CTCAE). After two cycles of therapy, the skin and joint disease progressed. Cobimetinib 20 mg PO daily on Days 1-21 every 28 days was then initiated. While he experienced improvement in his joint pain and immobility by Cycle 2, he suffered grade 3 acneiform eruptions (as defined by CTCAE) and intermittent severe abdominal pain without any other source. Dermatologist-directed acne therapy did not result in acceptable abatement. 

The patient was then started on clofarabine at 60 mg/m2 daily IV for five days every four weeks. However, this was held after two cycles due to grade 3 liver toxicity. 

After four cycles, clofarabine was stopped due to grade III nausea and lack of significant clinical improvement in skin lesions. Repeat FDG PET-CT scan showed areas of cutaneous thickening in the bilateral axillae (SUVmax 5.6) and perineal soft tissue (SUVmax 7.8). The regions of cutaneous thickening were also noted to be larger compared to previously.

**Figure 8:**
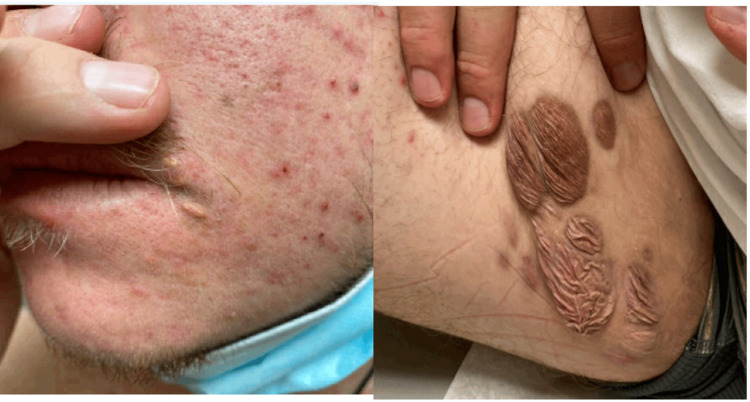
Skin lesions after four weeks of treatment with trametinib

**Figure 9:**
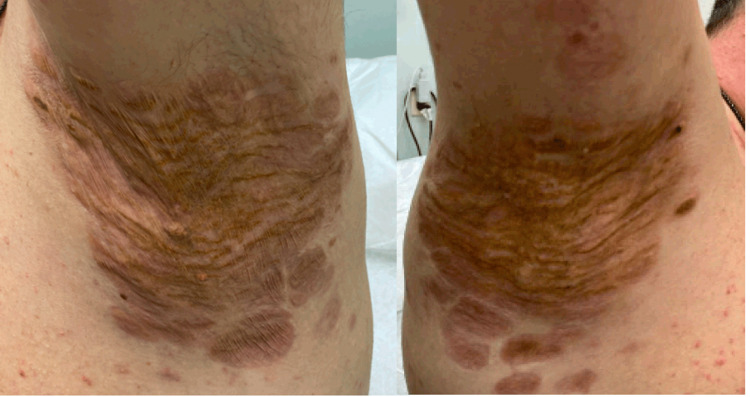
Skin lesions with four weeks of treatment with trametinib

For more consistent dosing, MEK inhibitor therapy with trametinib was started at 2 mg PO daily. One cycle resulted in significant improvement in the cutaneous xanthogranulomas (Figures 8-9).

However, uncontrollable acne again developed. Despite usual dermatologic interventions and a dose reduction to 1.5 mg daily, trametinib was discontinued after three cycles.

Fifth-line therapy with lenalidomide 20 mg PO daily on Day 1-21 every 28 days was then initiated. After two months of therapy, the affected joints and skin were stable. In order to achieve a more significant functional and cosmetic response, trametinib was resumed at 1 mg PO daily without change in lenalidomide dosing. 

**Figure 10:**
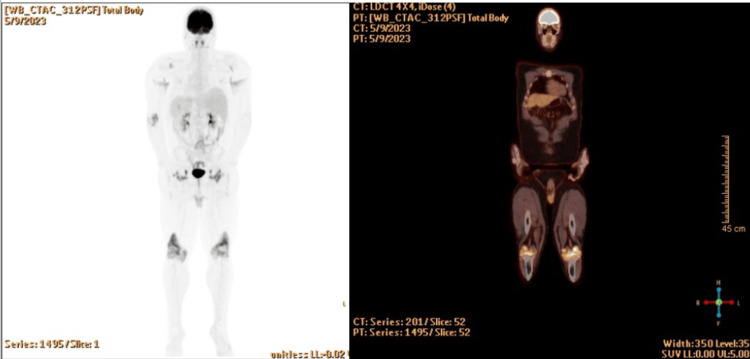
A repeat PET/CT scan shows hypermetabolism involving multiple joint spaces with associated bone erosion, as well as hypermetabolic cutaneous thickening in axillary and perineal regions

The patient tolerated the combination well with no significant side effects. Repeat FDG PET-CT showed FDG uptake involving multiple joint spaces again in the knees (SUVmax 15.2), hips (SUVmax 9.3), and elbows (SUVmax 6.4). FDG uptake was also seen in bilateral axillae with SUV max 4.7. which was improved from prior testing (Figure 10). 

**Figure 11:**
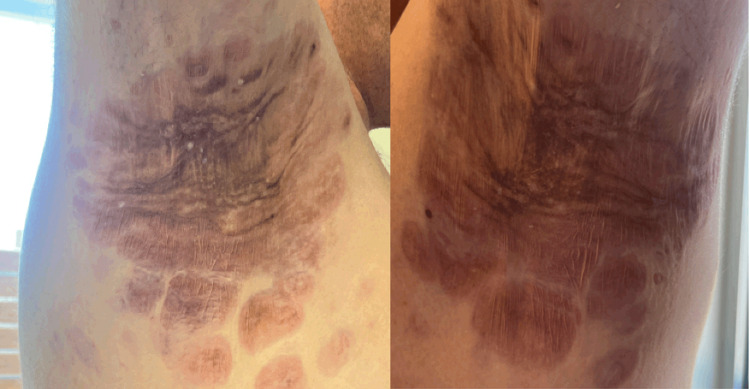
Skin lesions after seven months of treatment of lenalidomide and trametinib

A follow-up appointment with 12 cycles of the dual regimen showed significant improvement of his bilateral axillary lesions (SUVmax 4.2) and perineal region (SUVmax 3.8) (Figure 11). 

## Discussion

XD is a rare condition in which cutaneous lipid deposition occurs secondary to a proliferation of histiocytic cells [20,21]. Based on physical examination, the diagnosis of XD remains challenging due to the need to differentiate among eruptive xanthomas and other malignant histiocytoses such as Langerhans cell histiocytosis and Erdheim-Chester disease. Clinical correlation, specific histologic findings, and immunohistochemistry, tissue molecular studies, and highly sensitive imaging with MRI and FDG PET-CT are all required. 

There is no consensus in the medical literature regarding treatment for XD. Surgical excision and laser therapy have been shown to improve physical appearance, but the course of the disease is characterized by frequent relapses [22]. Treatment with antimitotic drugs has also been shown to be ineffective in many reported cases [23]. Oral steroids may offer some palliation and deter the recurrence of skin lesions after surgery. A case series by Khezri et al. showed response to 2-chlorodeoxyadenosine (cladribine), with five out of eight patients showing remission of their XD after a median of five cycles [22].

Importantly, cobimetinib treatment was shown to be efficacious regardless of the specific MAPK/ERK mutations the patients possessed [13]. However, while both MEK and BRAF inhibitors have been associated with high overall response rates in histiocytic neoplasms [13,14], immediate toxicity, the potential for delayed toxicity, and cost become increasingly important factors as continuous therapy appears to be necessary for a durable response [13, 24]. 

While this patient had classic cutaneous findings that developed at a typical age, he developed highly unusual synovial involvement that caused intractable pain and ambulation impairment. In addition to the need for cosmesis as his cutaneous manifestations were significantly disfiguring, including marked facial and perineal involvement, the therapeutic strategy focused additionally on the need to rapidly reduce pain and immobility. After disease progression and toxicities to typical agents, including corticosteroids, cladribine, cobimetinib, and clofarabine, he both tolerated and responded to dual therapy with lenalidomide and trametinib, with a time to response of approximately seven months.

While this patient had evidence for a clinically significant response to third-line therapy with cobimetinib, the eruption of acne, despite dermatologist-directed therapy, was therapy-limiting. Cladribine was intolerable due to refractory nausea and vomiting and was not rapidly effective. There is limited data on the safety of combining a MEK inhibitor with cladribine or clofarabine.

## Conclusions

Our patient with XD had previously received multiple lines of therapy, including cladribine, cobimetinib, and clofarabine, with limited efficacy and/or intolerable toxicity, reflecting the lack of an established standard of care for this rare histiocytic disorder.

In contrast, combination therapy with lenalidomide and trametinib led to sustained clinical improvement in both skin and joint lesions over 18 months. Ongoing monitoring is essential given the potential for ocular and cardiac toxicity from trametinib and the risk of secondary hematologic malignancies associated with both lenalidomide and non-LCH histiocytoses.

This case highlights the potential efficacy of MEK inhibitors combined with lenalidomide in XD, but further clinical studies are needed to define the optimal dosing and therapeutic role of this regimen.
